# High-Dose Dual Therapy Versus Bismuth Quadruple Therapy for Rescue Treatment of *Helicobacter pylori*: A Systematic Review and Meta-analysis of Randomized Controlled Trials

**DOI:** 10.5152/tjg.2026.25849

**Published:** 2026-03-13

**Authors:** Nan Lin, Jianxiong Liu, Changqing Yang

**Affiliations:** Department of Gastroenterology, The Affiliated Hospital of Putian University, Putian, China

**Keywords:** Bismuth-containing quadruple therapy, *Helicobacter pylori*, high-dose dual therapy, meta-analysis, rescue treatment

## Abstract

**Background/Aims:**

Failure of first-line *Helicobacter pylori* (HP) eradication therapy occurs in approximately 3%-24% of patients. High-dose dual therapy (HDDT) has been proposed as an alternative rescue regimen. This meta-analysis compared the efficacy and safety of HDDT and bismuth-containing quadruple therapy (BQT) in patients undergoing rescue eradication therapy.

**Materials and Methods:**

Randomized controlled trials (RCTs) comparing HDDT with BQT for HP rescue therapy were systematically searched in multiple databases from January 1, 2000, to June 30, 2025. Primary outcomes were eradication rates based on intention-to-treat (ITT) and per-protocol (PP) analyses. Secondary outcomes included treatment compliance and adverse events. Statistical analyses were performed using Stata 18.0 and RevMan 5.4.

**Results:**

Ten RCTs involving 2458 patients were included. In the ITT analysis, HP eradication rates were comparable between the HDDT and BQT groups (81.09% vs. 80.02%, relative risk (RR) = 1.01, 95% CI: 0.97-1.05, *P* = .54). Similarly, no significant difference was observed in the PP analysis (86.42% vs. 86.27%, RR = 1.00, 95% CI: 0.97-1.03, *P* = .90). However, HDDT was associated with significantly lower incidence of adverse events (14.00% vs. 35.13%, RR = 0.40, 95% CI: 0.31-0.51, *P* < .00001) and higher patient compliance (92.75% vs. 88.62%, RR = 1.08, 95% CI: 1.01-1.14, *P* = .02) compared with BQT.

**Conclusion:**

HDDT achieved eradication rates comparable to those of BQT for rescue therapy after initial treatment failure, with fewer adverse events and better patient compliance.

**Main Points**

- High-dose dual therapy (HDDT) showed eradication rates comparable to those of bismuth-containing quadruple therapy (BQT).
- HDDT was associated with fewer adverse events and improved compliance compared to BQT.
- HDDT is a well-tolerated option for the rescue treatment of *Helicobacter* *pylori* infection.

## Introduction

The human gastric mucosa is colonized by *Helicobacter pylori* (HP), a Gram-negative bacterium. The global prevalence of HP infection is approximately 60.3% and exceeds 80% in some developing countries.^1^ Peptic ulcer disease, gastric cancer, chronic gastritis, and gastric mucosa–associated lymphoid tissue lymphoma are gastrointestinal diseases closely associated with HP infection. In addition, HP has been associated with immune thrombocytopenic purpura and unexplained iron deficiency anemia, both recognized as extraintestinal manifestations.^2^ According to the Maastricht VI Consensus Report, HP infection is considered an infectious disease, and eradication therapy may be recommended in asymptomatic infected individuals, regardless of symptoms or complications.^3^

Current clinical guidelines underscore the importance of successful HP eradication with first-line therapy. Multiple therapeutic strategies have been recommended to improve HP eradication rates, including concomitant therapy, bismuth-containing quadruple therapy (BQT), hybrid therapy, sequential therapy, and vonoprazan-based therapy.^4 5^ However, first-line eradication therapy fails in 3%-24% of patients with HP.^5-7^ BQT is the primary regimen recommended by global consensus for rescue treatment of HP eradication.^8^ However, because of factors such as serious adverse drug effects, increasing antibiotic resistance, short treatment duration, poor patient adherence, and high cost, HP eradication rates with BQT have shown a declining trend.^9^ Antibiotic resistance during rescue therapy for HP infection predicts the outcomes of subsequent treatments. Clarithromycin, metronidazole, amoxicillin, levofloxacin, and tetracycline show HP resistance rates of 56%-71%, 35%-74%, 0%-8%, 21%-43%, and 0%-4%, respectively.^10-12^ Therefore, amoxicillin remains a key antibiotic for rescue therapy because of the persistently low resistance rate of HP.

High-dose dual therapy (HDDT) refers to the oral administration of amoxicillin and acid suppressants for 14 days, with a total amoxicillin dose of ≥3g/day and a dosing frequency of 3 to 4 times/day.^13^ In 1989, dual therapy for HP eradication was first described by Unge et al.^14^ However, when administered at standard doses and frequencies, eradication rates ranged from 55% to 75%, which were considered suboptimal. As a result, its clinical efficacy was limited, and the regimen was gradually replaced by other therapeutic strategies. By increasing the dosing frequency, dosage, and duration, recent studies have improved dual therapy. The HDDT, comprising amoxicillin combined with a proton pump inhibitor (PPI) or a potassium-competitive acid blocker (P-CAB), has shown modest efficacy. However, the effectiveness of HDDT as rescue treatment for HP remains controversial. A meta-analysis of studies comparing HDDT and BQT regimens for HP rescue treatment was conducted to assess the efficacy and safety of HDDT in this setting.

## Materials and Methods

### Search Approach

The study was conducted in accordance with the Preferred Reporting Items for Systematic Reviews and Meta-Analyses statement. The protocol was prospectively registered in the PROSPERO database (registration number: CRD420251086045). A comprehensive literature search was performed in the China National Knowledge Infrastructure, VIP Database, Wanfang Data, PubMed, Embase, the Cochrane Library, and Web of Science from January 1, 2000, to June 30, 2025, without language restrictions. The search strategy was as follows: (“amoxicillin”) AND (“dual therapy”) AND (“*Helicobacter pylori*” OR “*H.pylori*” OR “HP”). Ethical approval and informed consent were not required because all data were derived from previously published studies.

### Inclusion and Exclusion Criteria

Inclusion criteria were as follows: (1) patients with failure of first-line HP eradication who subsequently received rescue therapy; (2) studies in which the intervention group received HDDT; (3) studies in which the control group received BQT; (4) randomized controlled trial (RCT) design; and (5) reported outcomes including HP eradication rate, incidence of adverse events, and patient compliance. Exclusion criteria were as follows: (1) reviews, non-RCT studies, case reports, meta-analyses, editorials, conference abstracts, and animal studies; (2) studies involving first-line HP eradication therapy; (3) studies with interventions inconsistent with HDDT or BQT; (4) studies with incomplete outcome data; and (5) duplicate publications.

### Data Extraction

Two reviewers independently screened the retrieved studies according to predefined eligibility criteria. In cases of disagreement, a third author was consulted or a discussion was held to reach consensus. The extracted data included first author, publication year, region, study design, sample size, methods used to detect HP infection before and after eradication, timing of eradication confirmation, and specific treatment regimen (drug dosage, dosing frequency, and treatment duration). Extracted outcomes included eradication rates based on intention-to-treat analysis (ITT) and per-protocol analysis (PP) analyses, incidence of adverse events, and patient compliance. Compliance was defined as receipt of >80% of the recommended dosage. The primary outcome of the current study was HP eradication rate (ITT and PP), and secondary outcomes included the incidence of adverse events and patient compliance.

### Bias Risk Assessment

The Cochrane Risk of Bias Assessment Tool was used by the 2 authors to assess the risk of bias.^15^ The evaluated items included random sequence generation, allocation concealment, blinding of treatment and outcome assessment, comprehensiveness of outcome information, selective outcome reporting, and other sources of bias. Risk of bias for each item was classified as high, unclear, or low. Disagreements were resolved by consulting a third author or through discussion to reach consensus.

### Statistical Analysis

Statistical analyses were performed using RevMan version 5.4 (Nordic Cochrane Centre, Cochrane Collaboration; Copenhagen, Denmark). Relative risks (RRs) with 95% CIs were calculated for dichotomous outcomes. Heterogeneity among the included studies was assessed using the chi-square test and quantified with the *I*^2^ statistic. A fixed-effect model was used when heterogeneity was low (*I*^2^ ≤ 50% and *P* > .10), whereas a random-effects model was used when heterogeneity was high (*I*^2^ > 50% or *P* ≤ .10). A *P*value <.05 was considered statistically significant. Publication bias was assessed by visual inspection of funnel plots and further examined using Egger’s test in Stata version 18.0 (StataCorp, College Station; TX, USA). A *P*value <.05 was considered indicative of significant publication bias.

## Results

### Literature Search

**Figure 1.**
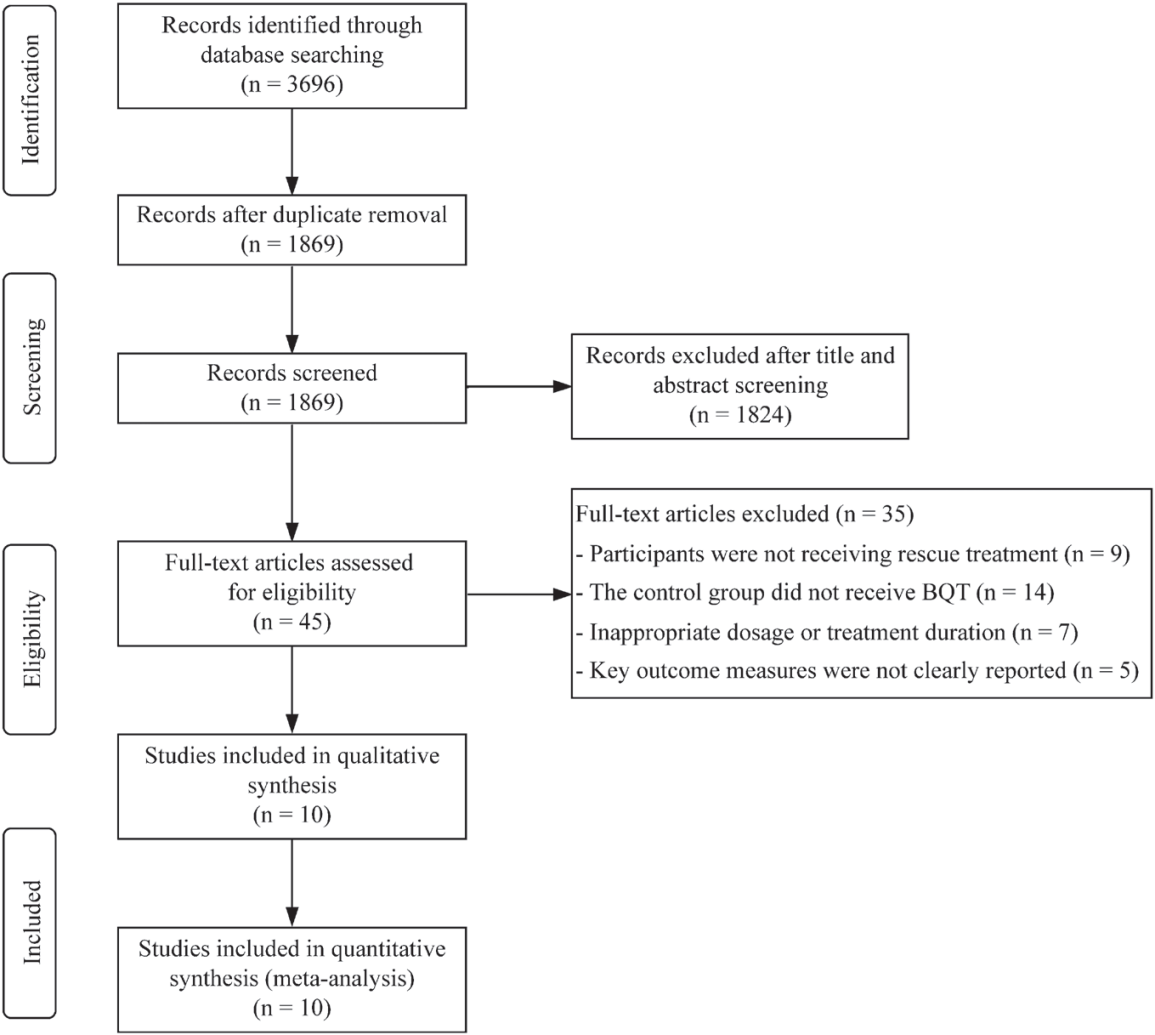
Literature search approach.

A total of 3696 articles were initially identified. After removal of 1827 duplicates, 1824 irrelevant articles were excluded based on title and abstract screening. Following full-text review, 35 articles were further excluded for the following reasons: participants not receiving rescue treatment (n = 9), absence of BQT in the control group (n = 14), inappropriate dosage or treatment duration (n = 7), and unclear reporting of key outcome measures (n = 5). Ultimately, 10 RCTs were included.^16-25^ The study selection process is illustrated in Figure 1.

### Characteristics of the Included Studies

The 10 included RCTs^16-25^ were published between 2003 and 2025. Of these, 8^17,19-25^ were conducted in mainland China, 1^18^ in Taiwan, China, and 1^16^ in Germany and Austria. Overall, the studies included 2458 patients, including 1232 in the HDDT group and 1226 in the BQT group. Table 1 presents the key features of the included studies.

### Risk of Bias

**Figure 2:**
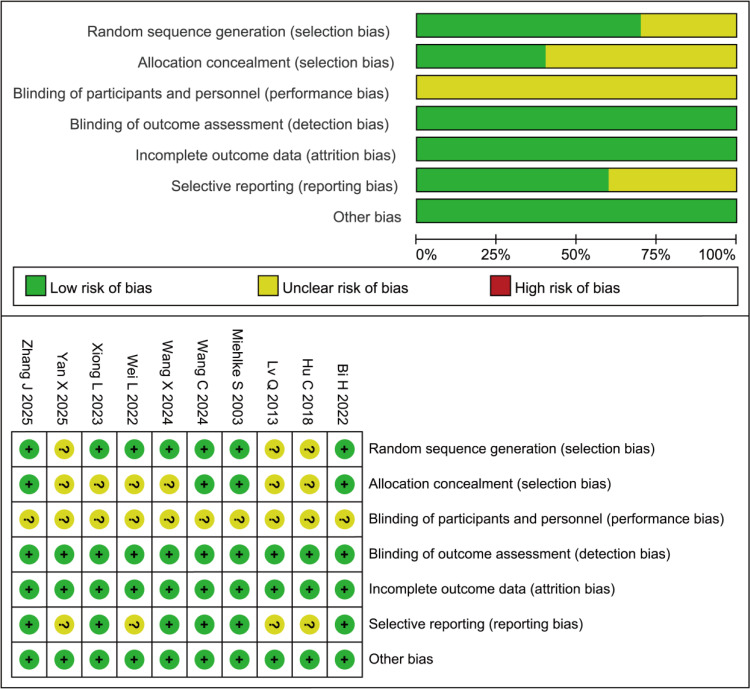
Risk of bias analysis chart.

Seven studies^16,19-23,25^ reported a clear method of random sequence generation, while 3 studies^17,18,24^ stated that randomization was performed but did not specify the method used. Four studies^16,19,22,25^ reported allocation concealment, whereas 6 studies^17,18,20,21,23,24^ did not provide detailed descriptions of the allocation concealment method. None of the 10 studies provided detailed information on blinding. Although blinding was not specified, this was judged unlikely to affect outcome measurement. The outcome measures were unlikely to be affected, although none of the 10 trials specified whether blinding was used for outcome assessment. All 10 trials reported complete outcome data, with no evidence of selective reporting, and no other sources of bias were identified. These findings are presented in Figure 2.

### *Helicobacter pylori *Eradication Rate

**Figure 3:**
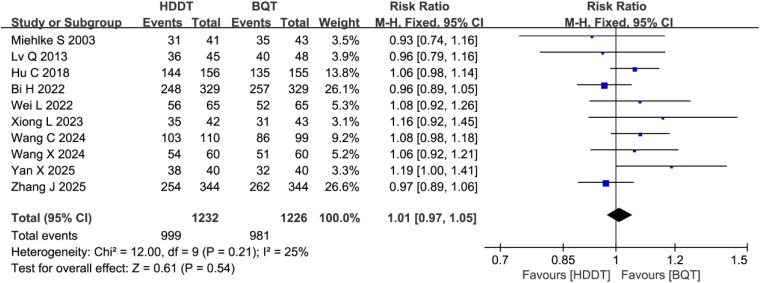
Forest plot of HP eradication rates (ITT).

**Figure 4:**
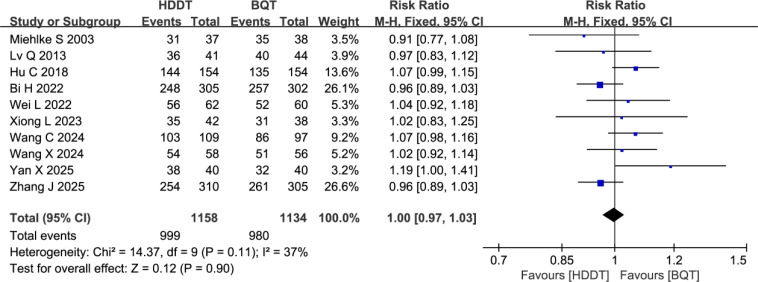
Forest plot of HP eradication rates (PP).

All 10 studies reported HP eradication rates for both HDDT and BQT. In the ITT analysis, eradication was achieved in 999 of 1232 patients in the HDDT group compared with 981 of 1226 patients in the BQT group. Low heterogeneity was observed (*I*^2^ = 25%, *P* = .21), and a fixed-effect model was used. No significant difference was found between the HP eradication rates of HDDT and BQT groups (81.09% vs. 80.02%, RR = 1.01, 95% CI: 0.97-1.05, *P* = .54) (Figure 3). In the PP analysis, eradication was achieved in 999 of 1158 patients in the HDDT group, compared with 980 of 1134 patients in the BQT group. Low heterogeneity was observed (*I*^2^ = 37%, *P* = .11) and a fixed-effect model was used. No significant difference was found between the HP eradication rates of HDDT and BQT groups (86.27% vs. 86.42%, RR = 1.00, 95% CI: 0.97-1.03, *P* = .90) (Figure 4).

### Subgroup Analysis of *Helicobacter pylori* Eradication Rates by Acid-Inhibitor Type in the High-Dose Dual Therapy Regimen

**Figure 5:**
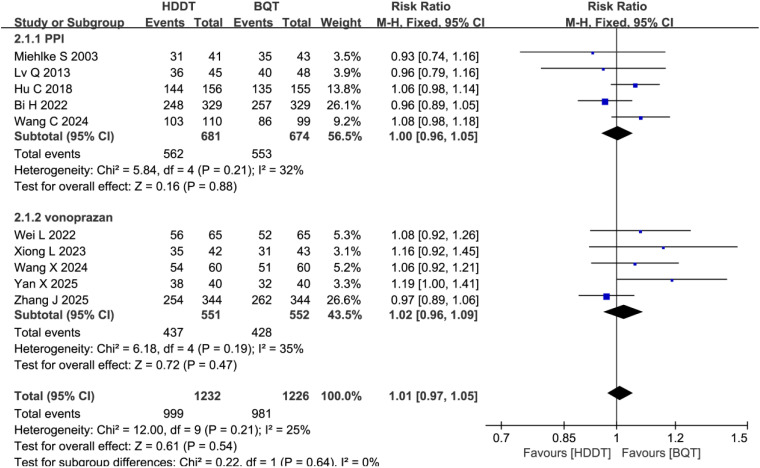
Forest plot of subgroup analysis of HP eradication rates (ITT).

**Figure 6:**
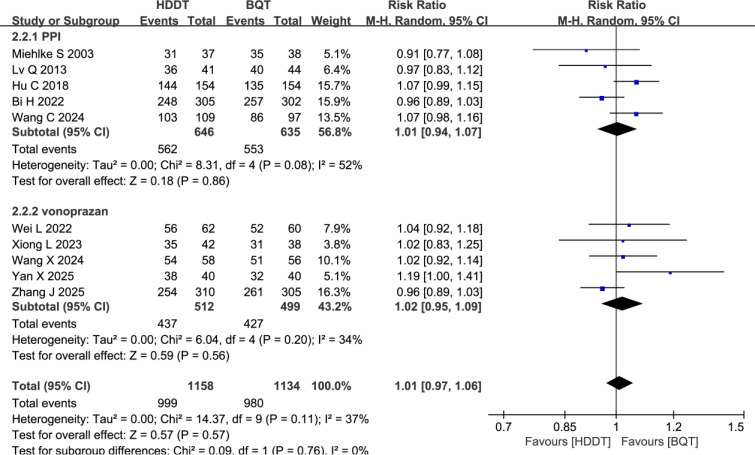
Forest plot of subgroup analysis of HP eradication rates (PP).

Among the 10 studies, 5 studies^16-19,22^ used PPIs as the acid-inhibitor agents in the HDDT regimen, whereas the remaining 5^20,21,23-25^ used vonoprazan. A subgroup analysis was performed according to acid-inhibitor type in the HDDT regimen. In the ITT analysis, HP eradication rates did not differ significantly between the HDDT and BQT groups in the PPI-based subgroup (82.53% vs. 82.05%, RR = 1.00, 95% CI: 0.96-1.05, *P* = .88). Similarly, no significant difference was observed in the vonoprazan-based subgroup (79.31% vs. 77.54%, RR = 1.02, 95% CI: 0.96-1.09, *P* = .47) (Figure 5). In the PP analysis, HP eradication rates also did not differ significantly between the HDDT and BQT groups in the PPI-based subgroup (87.00% vs. 87.09%, RR = 1.01, 95% CI: 0.94-1.07, *P* = .86). Likewise, no significant difference was observed in the vonoprazan-based subgroup (85.35% vs. 85.57%, RR = 1.02, 95% CI: 0.95-1.09, *P* = .56) (Figure 6).

### Adverse Events

**Figure 7:**
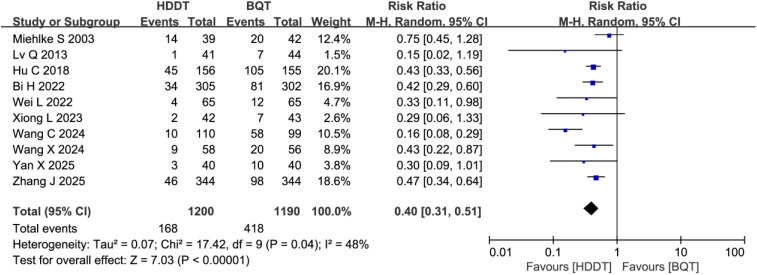
Forest plot of adverse events

Adverse events were reported in all 10 trials. A comprehensive summary of adverse events across the included studies was prepared. Patients in both groups experienced adverse events, including nausea, taste disturbances, abdominal distension, diarrhea, dizziness, headache, and abdominal pain. No serious adverse events were reported, and all adverse events gradually resolved after discontinuation of the drug. Heterogeneity between the groups was observed (*P* = .04, *I*^2^ = 48%), and a random-effects model was used. The HDDT group had fewer adverse events than the BQT group (14.00% vs. 35.13%, RR = 0.40, 95% CI: 0.31-10.51, *P* < .00001) (Figure 7).

### Patient Compliance

**Figure 8:**
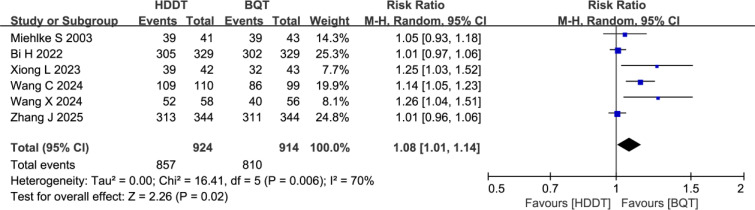
Forest plot of patient compliance.

A total of 6 studies^16,19,21-23,25^ reported patient compliance. Significant heterogeneity was observed (*I*^2^ = 70%, *P* = .006), and a random-effects model was used. The HDDT group had higher patient compliance than the BQT group (92.75% vs. 88.62%, RR = 1.08, 95% CI: 1.01-1.14, *P* = .02) (Figure 8).

### Sensitivity Analysis

**Figure 9:**
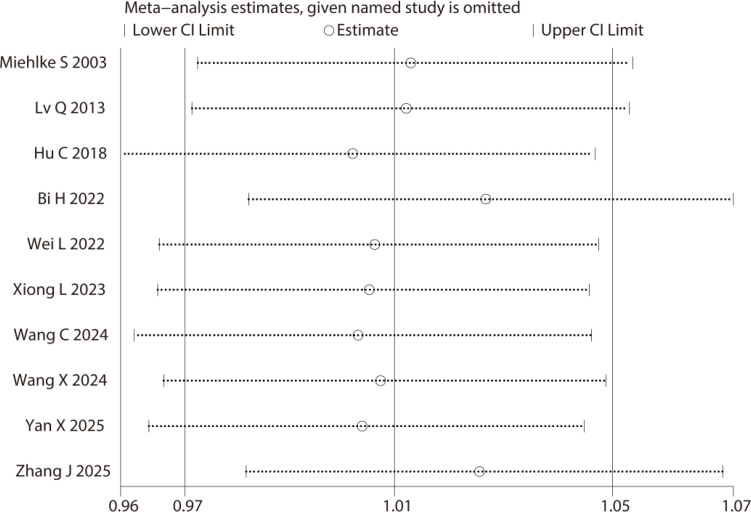
Sensitivity analysis of HP eradication rate (ITT).

The leave-one-out approach was used to perform sensitivity analysis for HP eradication rate (ITT). Exclusion of any single study had a negligible effect on the overall results, with only minor changes in heterogeneity, indicating that the findings were robust (Figure 9).

### Publication Bias

**Figure 10:**
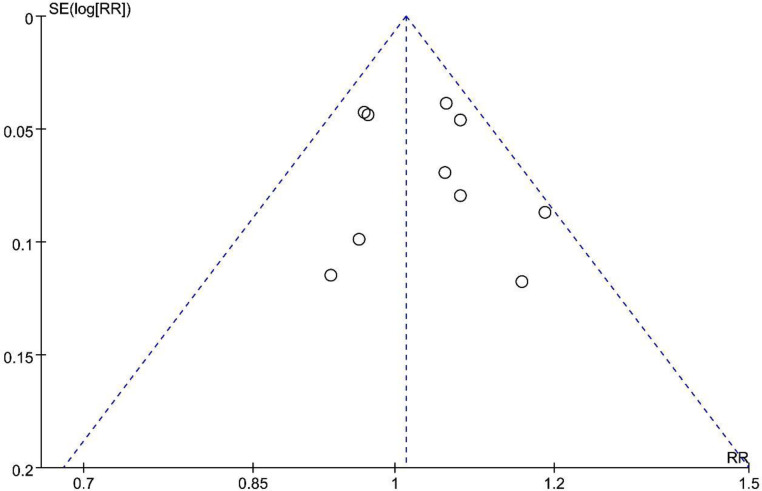
Funnel plot of HP eradication rate (ITT).

**Figure 11:**
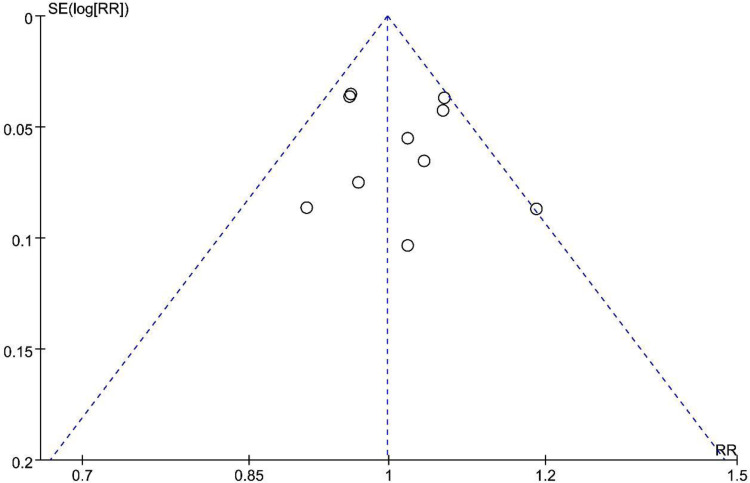
Funnel plot of HP eradication rate (PP).

**Figure 12:**
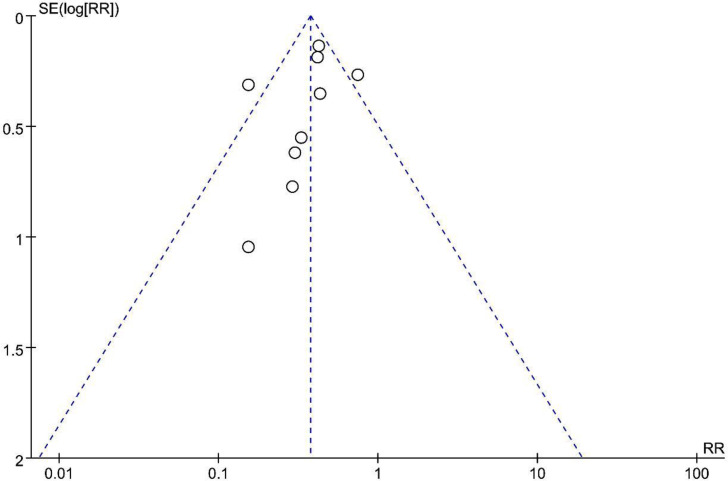
Funnel plot of adverse events.

Publication bias was assessed by qualitative evaluation of funnel plots and quantitative analysis using Egger’s test when more than 10 studies were included in the meta-analysis. The *P* values for the HP eradication rate were .54 in the ITT analysis and .63 in the PP analysis. The *P* value for adverse events was .29, indicating no evidence of significant publication bias (Figures 10-12).

## Discussion

This meta-analysis compared the efficacy, safety, and patient compliance of HDDT and BQT regimens in HP rescue therapy. The findings of the current study showed that, as a rescue regimen for HP infection after initial treatment failure, HDDT achieved eradication rates comparable to those of BQT, with fewer adverse events and better compliance. Subgroup and sensitivity analyses confirmed the robustness of these findings, and no evidence of significant publication bias was observed.

International consensus currently recommends BQT as both first-line and second-line therapy for HP eradication. However, because of factors such as increasing antibiotic resistance, poor patient compliance, insufficient treatment duration, and high treatment costs, the success rate of BQT as a first-line eradication therapy has gradually declined.^26^ If antibiotics with high resistance rates, such as levofloxacin, metronidazole, and clarithromycin, were used in the initial regimen, they should be avoided in the subsequent rescue treatment.^27^ After failure of first-line HP eradication treatment, the available choice of antibiotics for rescue treatment becomes further limited. If subsequent eradication therapy also fails, antimicrobial resistance may worsen, requiring more complex treatment strategies and increasing future medical costs. Therefore, treatment after initial therapeutic failure remains challenging and is a major concern for clinicians.

Previous studies comparing the eradication efficacy of HDDT with other treatment regimens for HP rescue therapy have reported inconsistent results. In a prospective randomized controlled study of patients who had failed first-line HP treatment, Yang et al^28^ reported that the HDDT group achieved higher eradication rates than both the clarithromycin-based triple therapy and the sequential therapy groups. However, in another randomized controlled study of rescue therapy in patients who had failed first-line HP treatment, Okimoto et al^29^ reported no significant difference in eradication rates between the levofloxacin-based triple therapy and the HDDT groups. These discrepancies were attributed to differences in the comparator regimens across studies. Therefore, in this meta-analysis, we selected BQT, recommended by international consensus guidelines and widely used in clinical practice, was selected as a uniform comparator.

Despite the widespread use of amoxicillin in HP eradication therapy over many years, HP resistance to amoxicillin has remained low.^30^ For HP to develop resistance to amoxicillin, multiple mutations in several penicillin-binding protein–related gene sites are required, which may explain the low resistance rate to amoxicillin.^31^ Therefore, given the high resistance rates to other antibiotics, amoxicillin remains the preferred agent in dual therapy regimens.

Amoxicillin is a time-dependent antibiotic that is rapidly absorbed and eliminated within 6-8 hours.^32^ The bactericidal activity of amoxicillin depends on the duration for which its plasma concentration exceeds the minimum inhibitory concentration. Therefore, increasing the daily dose and administering the drug 3 to 4 times daily can enhance its bactericidal activity.^33^ When gastric pH exceeds 6.0, HP proliferates actively and becomes more susceptible to amoxicillin. However, when the pH is below 6.0, HP undergoes coccoid transformation, reducing antibiotic efficacy.^34^ Antibiotics are unstable in acidic environments. The use of acid inhibitors to increase gastric pH can improve the stability and bioavailability of antibiotics in the stomach.^35^

Currently, the main acid inhibitors used in HP eradication therapy are PPIs and P-CABs. PPIs are the most widely used acid agents, with a half-life of 0.5-2.1 hours. Increasing the dosing frequency of PPIs helps maintain effective acid suppression.^36^ The metabolism of PPIs is influenced by CYP2C19gene polymorphisms; therefore, increasing PPI dosing frequency helps achieve effective acid suppression across different CYP2C19.^37^ The P-CABs, such as vonoprazan, are increasingly used in clinical practice for HP eradication therapy as a novel class of acid inhibitors. Compared with traditional PPIs, vonoprazan provided stronger, more stable, and longer-lasting acid suppression. It does not require an enteric coating, remains stable in acidic environments, and is not affected by CYP2C19 gene polymorphisms or food intake.^38^ Compared with BQT, dual therapy with P-CAB and amoxicillin yielded higher eradication rates in first-line HP therapy.^39^ In the current study, subgroup analysis showed that, for rescue treatment of HP infection, HDDT achieved eradication rates comparable to those of BQT, regardless of whether the acid inhibitor was a PPI or a P-CAB.

According to the criteria proposed by the internationally recognized gastroenterologist Graham DY, the acceptable lower limits for HP eradication success are 80% for ITT analysis and 85% for PP analysis.^38^ In this meta-analysis, eradication rates of HDDT were only slightly above these thresholds, and in regimens using vonoprazan as the acid inhibitor, the HP eradication rates in the ITT analysis were even slightly below 80%. These findings indicate that, although HDDT demonstrated efficacy comparable to that of BQT, overall eradication rates in rescue therapy remain suboptimal. Notably, rescue therapy is administered after failure of initial treatment. In this context, bacterial resistance is more common and may reduce treatment efficacy. These findings further highlight the need to optimize drug dosage and dosing frequency based on antimicrobial resistance patterns to improve HP eradication rates in rescue therapy.

Regarding adverse events, those associated with BQT included nausea, taste disturbances, abdominal distension, diarrhea, dizziness, headache, and abdominal pain, which were mainly related to the combined use of antibiotics and bismuth compounds.^40^ In terms of adverse event profiles, the HDDT and the BQT groups were generally comparable. Despite increased dosing frequency and cumulative daily doses of acid inhibitors and amoxicillin, the HDDT group had fewer adverse events than the BQT group. Most of these events were mild to moderate, self-limited, resolved after treatment, and did not require specific intervention.

Patient compliance may be influenced by several factors, including adverse drug reactions, the complexity of the treatment regimen, inadequate patient education provided by health care professionals, and limited patient understanding of the disease, all of which can affect treatment outcomes. The higher compliance observed with the HDDT group compared with the BQT group may be attributed to its simpler dosing regimen and the lower incidence of adverse events.

This meta-analysis has several limitations. First, potential bias exists because none of the included studies were double-blind trials. Second, most included studies were conducted in China, with only 1 study performed in Germany and Austria. This geographic concentration may limit the generalizability of the findings, as variations in antibiotic resistance patterns and baseline eradication rates across countries could influence treatment efficacy. Therefore, these results should be interpreted with caution in the context of local epidemiological characteristics and should not replace region-specific clinical guidelines. Third, some heterogeneity was observed in the secondary outcomes of patient compliance and adverse events. Fourth, the analysis included only a limited number of studies; therefore, additional multicenter, large-scale studies are needed to validate these findings. Future research should address other issues, such as treatment costs.

In conclusion, this meta-analysis showed that HDDT achieved eradication rates comparable to those of BQT in patients receiving rescue treatment after failed initial HP therapy, with fewer adverse events and higher patient compliance.

## Figures and Tables

**Table 1.** Main Features of the Included RCTs

**Author**	**Publication Year**	**Country**	**Study Design**	**Patients (stu/con) (n)**	**Test to Diagnose HP**	**Time to Confirm Eradication**	**Test to Confirm Eradication**	**Study Regimen**	**Control Regimen**
Miehlke S^16^	2003	Germany and Austria	Multicenter RCT	84 (41/43)	H/C	6-8 weeks	H/C/UBT/RUT	Ome 40 mg qid, Amo 750 mg qid, 14 days	Ome 20 mg bid, Bis 107 mg qid, Met 500 mg qid, Tet 500 mg qid, 14 days
Lv Q^17^	2013	Chinese mainland	Single-center RCT	93 (45/48)	UBT/RUT	4 weeks	UBT	Rab 20 mg bid, Amo 1000 mg tid, 14 days	Rab 10 mg bid, Bis 220 mg bid, Fur 100 mg bid, Ruf 200 mg qd, 14 days
Hu C^18^	2018	Taiwan, China	Multicenter RCT	311 (156/155)	H/C	4-8 weeks	UBT	Rab 20 mg qid, Amo 750 mg qid, 14 days	Rab 20 mg bid, Bis 300 mg qid, Met 250 mg qid, Tet 500 mg qid, 10 days
Bi H^19^	2022	Chinese mainland	Multicenter RCT	658 (329/329)	H/UBT/C/SAT	4-8 weeks	UBT/SAT	Eso 40 mg tid, Amo 1000 mg tid, 14 days	Eso 40 mg bid, Bis 220 mg bid, Fur 100 mg bid, Tet 500 mg tid, 14 days
Wei L^20^	2022	Chinese mainland	Single-center RCT	130 (65/65)	UBT	4 weeks	UBT	Von 20 mg bid, Amo 1000 mg tid, 14 days	Eso 20 mg bid, Bis 220 mg bid, Amo 1000 mg bid, Fur 100 mg bid, 14 days
Xiong L^21^	2023	Chinese mainland	Single-center RCT	85 (42/43)	UBT	4 weeks	UBT	Von 20 mg bid, Amo 1000 mg tid, 14 days	Eso 20 mg bid, Bis 220 mg bid, Amo 1000 mg bid, Fur 100 mg bid, 14 days
Wang C^22^	2024	Chinese mainland	Multicenter RCT	209 (110/99)	UBT	4 weeks	UBT	Eso 20 mg qid, Amo 750 mg qid, 14 days	Eso 20 mg bid, Bis 240 mg bid, Amo 1000 mg bid, Fur 100 mg bid, 14 days
Wang X^23^	2024	Chinese mainland	Single-center RCT	120 (60/60)	UBT	4 weeks	UBT	Von 20 mg bid, Amo 1000 mg tid, 14 days	Rab 20 mg bid, Bis 200 mg bid, Amo 1000 mg bid, Fur 100 mg bid, 14 days
Yan X^24^	2025	Chinese mainland	Single-center RCT	80 (40/40)	UBT	4 weeks	UBT	Von 20 mg bid, Amo 750 mg qid, 14 days	Rab 20 mg bid, Bis 220 mg bid, Amo 1000 mg bid, Fur 100 mg bid, 14 days
Zhang J^25^	2025	Chinese mainland	Multicenter RCT	688 (344/344)	H/C/RUT/SAT	4 weeks	UBT/SAT	Von 20 mg bid, Amo 1000 mg tid, 14 days	Eso 20 mg bid, Bis 220 mg bid, Fur 100 mg bid, Tet 500 mg tid, 14 days

Amo, amoxicillin; bid, twice daily; Bis, bismuth; C, biopsy culture; con, control group (BQT); Eso, esomeprazole; Fur: Furazolidone; H, histological examination; HP, *Helicobacter pylori*; Met, metronidazole; Ome, omeprazole; qd, once daily; qid, 4 times daily; Rab, rabeprazole; RCT, randomized controlled trial; Ruf, rufloxacin; RUT, rapid urea test; SAT, stool antigen test; stu, study group (HDDT); Tet, tetracycline; tid, thrice daily; UBT, urea breath test; Von, vonoprazan.

## Data Availability

The data that support the findings of this study are available on request from the corresponding author.
